# Cytologic findings of peripheral T-cell lymphoma (PTCL) with high epitheloid cell content (Lennert's lymphoma) in imprint smear. A case report

**DOI:** 10.1186/1742-6413-3-3

**Published:** 2006-02-06

**Authors:** Yahya Daneshbod

**Affiliations:** 1 Department of Hematopathology, University of Kiel, and Lymph Node Registry, Kiel, Germany

## Abstract

**Background:**

Lymphoepitheloid lymphoma is a T-cell lymphoma with peculiar histologic picture with massive proliferation of epitheloid cell clusters intermingled with many, mostly small-sized lymphoid cells. The cytomorphologic features in imprint of a case of lymphoepitheloid T-cell lymphoma is described together with diagnostic pitfalls.

**Case report:**

A 74 years old man presented with weight loss, anemia and fever. Physical examination showed bilateral cervical lymphadenopathy with hepatosplenomegaly. With the clinical impression of hypersplenic syndrome vs lymphoma, excisional biopsy of a lymph node was performed and both imprints and histologic sections made. Cytologic findings showed uniform isolated small lympocytes with closely intermingled scattered and aggregates of epitheloid cells. Histologic sections were diagnosed as lymphoepitheloid lymphoma (Lennert's lymphoma).

**Conclusion:**

Cytologic findings of this variant of lymphoma is distinctive enough for a correct initial suggestive diagnosis. However the presence of high content of epitheloid cell clusters can cause cytologic misinterpretation with other benign and malignant conditions.

## Background

Fine needle aspiration (FNA) and touch imprint cytology is widely used in the intial investigation of lymphadenopathy, and offers immediate preliminary diagnosis. Lymphoepitheloid lymphoma (Lennert lymphoma) is a particular variant of peripheral T- cell lymphoma with almost unique histologic morphology characterized by two predominant cell populations: epitheloid cells and T lympocytes, mainly of T helper/inducer phenotype[2].

However other benign or malignant conditions can partly mimic this picture [5]. Cytomorphology of this variant of lymphoma has been very rarely described[10]. So we like to describe the cytologic findings of this variant of peripheral T- cell lymphoma together with diagnostic pitfalls.

## Case report

In 1982, a 74 year old diabetic man presented with weight loss, anorrhexia and fever. Physical examination showed hepatosplenomegaly. Laborotory findings were a Hb: 9.4 g/d1, WBC: 1800/ml, PLT: 180,000, low albumin with normal serum iron. With clinical impression of hypersplenism vs lymphoma, lymph node excisional biopsy and imprint smear were made.

**Figure 1:**
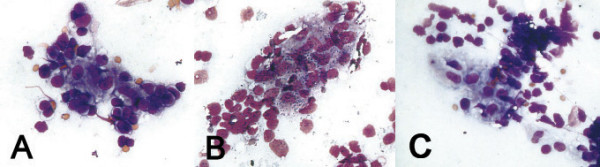
ABC: Small and large groups and clusters of epitheloid cells intermingled with the lymphocytes (Pappenheim × 400, ×400, ×400).

**Figure 2:**
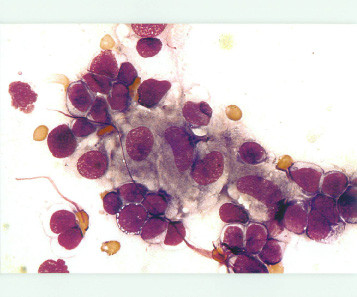
Epitheloid histiocytes showed oval to bean shaped nucleus with finely dispersed chromatin, twice the size of the lymphocytes with ample grayish granular cytoplasm (Pappenheim × 1000).

Cytologic smears were air-dried and stained by Pappenheim. Pappenheim stain is equivalent to Wright-Gimsa/ Diff- Quick stain. The smear was cellular and contained mainly (monomorphic) population of lymphocytes with round to oval nucleus and fine, evenly dispersed chromatin. Cytoplasm were light blue to clear. Rare cells with nuclear irregularity were noted. There were also small and large groups and clusters of epitheloid histiocytes intermingled with the lymphocytes (Fig. 1ABC). Epitheloid histiocytes shows oval to bean shaped nucleus with finely dispered chromatin, twice the size of the lymphocytes with ample grayish granular cytoplasm(Fig. 2). However no distinct granuloma were noted. Multinucleated giant cell or lymphogranular bodies were not noted.

**Figure 3:**
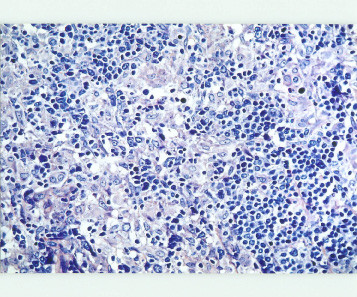
Sections of the lymph node showed a completely effaced architecture with exuberant proliferation of epitheloid cells in cluster and sheets, intermingled with small round to oval neoplastic lymphocytes (Giemsa, ×400).

Histologic sections of the lymph node showed a completely effaced architecture with exuberant proliferation of histiocytes in cluster and sheets, intermingled with small round to oval neoplastic lymphocytes (Fig 3). The final histologic report was "Lennert's lymphoma, with possibility of immunocytoma and epitheloid rich Hogkins disease, however cytomorphology is against Hogkins disease"(histologic diagnosis made by K. Lennert in Kiel, Germany, 1982). Immunohistochemical study (markers done not known) was in favor of an epitheloid rich T cell lymphoma. Reanalysis of stored paraffin blocks from 1982 showed an immunophenotype typical of peripheral T cell lymphoma with strong uniform positivity for CD5 and CD4 (Fig. 5) and negative for CD8, CD20. Gene rearrargement for T cell receptor was performed which confirmed a monoclonal process. Bone marrow biopsy was done and reported as extensive infiltration by malignant lymphoma cells with remarkable presence of macrophages and epitheloid cells with fibrosis (histologic diagnosis made by Lutz Dietrich Leder in Kiel, Germany, 1982) which he could not classify but also thought of hairy cell leukemia.

## Discussion

Lymphoepitheloid T- cell was first described by Lennert (Habilitation thesis, 1952) and published (1968)[1], and later widely known as Lennert's lymphoma. Older individuals are generally affected. Splenomegaly and lymphadenopathy may be prominent features, with involvement of Waldayer's ring also observed in European cases [7]. However in Revised European-American classification of lymphoid neoplasms (R.E.A.L) and World Health Organization (W.H.O), it is included in the peripheral T-cell lymphoma not otherwise specified (NOS)[4]. Histologically lymphoepitheloid cell variant (Lennert lymphoma) shows diffuse or (more rarely) interfollicular infiltrates consisting predominantly of small cells with only slight nuclear irregularities. Numerous clusters of epitheloid histocytes are present [4]. Clear cells or high endothelial venules are less frequent than in peripheral T- cell lymphomas of angioimmunoblastic or T-zone type. Few Reed-Sternberg-like cells, eosionophils and plasma cells can be seen [4]. However in our case they were absent on histology and cytology. Another term which should not be confused with this lymphoma is the Lennert's pattern. This is a histologic picture of diffuse scattered epitheloid population throughout the lymph node [5]. Differential diagnosis of Lennert's pattern includes both benign and malignant conditions such as: granulomatous lymphadenitis, tuberculosis, sacrocoidosis, abnormal immune response, peripheral T-cell lymphoma, T-cell rich B-cell lymphoma, mixed cellularity Hodgkin lymphoma, nodular lymphocytic and histiocytic Hodgkin's lymphoma and lymphoepithelioma- like carcinoma [5,8]. Cytologically all the mentioned diseases can partly simulate lymphoepitheloid lymphoma. In this case aggregates of epitheloid cell closely mimicked granuloma in which conditions like TB should be considered. However this case showed no multinucleated giant cell or polymorphic background. The monomorphic back ground can easily rule out sarcoidosis or othe reactive or immunologic process. The cytology of natural Killer/T-cell lymphomas show more pleomorphism with irregular chromatin distribution and usually no epitheloid cells [1]. FNA cytology of lymph node involvement by high grade mycosis fungoides with monotonous population of large and small cells can be misinterpreted with our case. However mycosis shows more anisocytosis with cerebriform convoluted nuclei against a background of atypical lymphocytes, and plasma cells [3,6]. Those cases of T- cell rich B- cell lymphoma (TCRBL) which can show also many epitheloid cells and predominance of mature small lymphocytes with few or absent atypical large, immature lymphoid cells should be considered in differential diagnosis [9]. However atypical large lymphoid cells are much more scarce in Lennert's lymphoma and immunocytochemistry can clearly differentiate these cases, which the large cells show B-cell marker and the small lymphocytes does not have aberrant antigen expression. To our knowledge, literature on cytologic findings of lymphoepitheloid lymphoma is sparse[10]. Although final diagnosis of this variant of T cell lymphoma is confirmed by immunohistochemical studies, cytologically the presence of monomorphic lymphoid background with mild to absent atypia with closely intermingled clusters of epitheloid histiocytes help for a correct preliminary working diagnosis.

**Figure 4:**
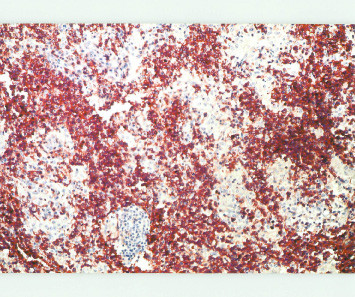
Uniform positivity for CD5 in lymphoid cells (×400).
